# The role of genetic sequencing and analysis in the polio eradication programme

**DOI:** 10.1093/ve/veaa040

**Published:** 2020-05-20

**Authors:** David Jorgensen, Margarita Pons-Salort, Alexander G Shaw, Nicholas C Grassly

**Affiliations:** Department of Infectious Disease Epidemiology, Imperial College London, St Mary's Campus, Norfolk Place, London W2 1PG, UK; Department of Infectious Disease Epidemiology, Imperial College London, St Mary's Campus, Norfolk Place, London W2 1PG, UK; Department of Infectious Disease Epidemiology, Imperial College London, St Mary's Campus, Norfolk Place, London W2 1PG, UK; Department of Infectious Disease Epidemiology, Imperial College London, St Mary's Campus, Norfolk Place, London W2 1PG, UK

**Keywords:** RNA virus, poliovirus, phylogenetics, eradication

## Abstract

Genetic sequencing of polioviruses detected through clinical and environmental surveillance is used to confirm detection, identify their likely origin, track geographic patterns of spread, and determine the appropriate vaccination response. The critical importance of genetic sequencing and analysis to the Global Polio Eradication Initiative has grown with the increasing incidence of vaccine-derived poliovirus (VDPV) infections in Africa specifically (470 reported cases in 2019), and globally, alongside persistent transmission of serotype 1 wild-type poliovirus in Pakistan and Afghanistan (197 reported cases in 2019). Adapting what has been learned about the virus genetics and evolution to address these threats has been a major focus of recent work. Here, we review how phylogenetic and phylogeographic methods have been used to trace the spread of wild-type polioviruses and identify the likely origins of VDPVs. We highlight the analysis methods and sequencing technology currently used and the potential for new technologies to speed up poliovirus detection and the interpretation of genetic data. At a pivotal point in the eradication campaign with the threat of anti-vaccine sentiment and donor and public fatigue, innovation is critical to maintain drive and overcome the last remaining circulating virus.

## 1. Introduction

Poliovirus is a species C Enterovirus, with three serotypes (one, two, and three). The virus has only one natural host, humans, and is primarily spread between individuals by faecal-oral transmission. In rare cases poliovirus can enter the central nervous system, leading to paralytic poliomyelitis (acute flaccid paralysis (AFP)). Although genomics was in its infancy when work on preventing poliomyelitis began, poliovirus was one of the earliest genomes to be sequenced in full, with two groups publishing serotype one sequences in 1981 (Kitamura et al. 1981; Racaniello and Baltimore 1981). Following the certification of smallpox eradication in 1980, and with rapid advancements towards disease control and elimination using efficacious vaccines, poliovirus was selected as a World Health Organization (WHO) eradication target with a global campaign starting in 1988 led by the Global Polio Eradication Initiative (GPEI). 

The pre-GPEI history of efforts to understand poliovirus biology have been described in detail previously (Koch and Koch 1985). Following early failed attempts at vaccine development, successful non-neuronal culture of serotype two poliovirus was achieved in 1949, a major step towards the production of the current vaccines (Enders, Weller, and Robbins 1949). In the same year, the three serotypes were identified and subsequently codified by the Committee on Typing of the National Foundation of Infantile Paralysis (USA) in 1951 (Bodian, Morgan, and Howe 1949; Committee on Typing of National Foundation of Infantile Paralysis 1951). With *in vitro* poliovirus growth a possibility, and knowledge of the three serotypes, successful development of inactivated poliovirus vaccine (IPV) and live-attenuated oral poliovirus vaccine (OPV) followed in 1953 and 1956, respectively (Salk 1953; Sabin 1956).

With a cheap, effective, and orally administered vaccine available and a virus with no known animal reservoir, eradication seemed like an imminent possibility when the GPEI was launched (World Health Organization 1988; John and Dharmapalan 2019). At that time, approximately 1,000 people a day were paralysed by wild poliovirus (WPV) (Bandyopadhyay et al. 2015). This has since been reduced by over 99.9 per cent, with WPV serotypes two and three eradicated in 2000 and 2012, respectively, and circulation of wild serotype one (WPV1) limited to Pakistan and Afghanistan. Despite this progress, prolonged inaccessibility of children to immunisation programmes, political turmoil, and anti-vaccine sentiment over the last few years has seen WPV1 cases climb from 28 in 2016 to 163 in 2019. The persistence of WPV1 in politically unstable and inaccessible regions is a WHO Public Health Emergency of International Concern (PHEIC) due to the threat of international spread (World Health Organization 2014b).

Another major hurdle to eradication is the emergence and spread of vaccine-derived polioviruses (VDPVs), which are OPV strains (often also called Sabin strains, in reference to the researcher who led the development of OPV) that have regained transmissibility and pathogenicity and can cause outbreaks of paralysis similar to their wild counterparts (Jenkins et al. 2010). Although the overall net benefit from the use of attenuated virus vaccines has been huge over the course of the polio eradication campaign, the occurrence of VDPV outbreaks in recent years has highlighted the need to phase out OPV. This transition started with the withdrawal of serotype 2 OPV during a globally coordinated ‘switch’ from trivalent to bivalent OPV in over 150 countries in April 2016. To mitigate risks associated with this withdrawal of vaccine, WHO recommended that at least one dose of IPV be given to infants during routine immunisation (World Health Organization 2017). However, a single dose of IPV results in seroconversion in just 50 − 80 per cent of children (depending on the age at administration; Grassly 2014; Bandyopadhyay et al. 2018) and its delayed introduction as a result of supply shortages and limited coverage has resulted in a growing cohort of children unimmunised against serotype two poliovirus. These children are exposed to an increasing incidence of serotype two VDPV (VDPV2), which has emerged both from trivalent OPV (tOPV) used before the switch and more recently as a result of monovalent serotype two OPV (mOPV2) used to control VDPV outbreaks (Macklin et al. 2020). In 2019, there were 270 confirmed VDPV2 paralysis cases across Africa, the Middle East and south Pacific, with 15 countries affected (World Health Organization 2020). 

This review describes how collection and analysis of increasing amounts of genetic data is transforming the long-standing poliovirus eradication programme, highlighting both current activities and future promise. It emphasises how our understanding of poliovirus genetics and evolution will be critical to success in tackling the major challenges facing the GPEI, allowing us to better describe transmission of virus and develop more efficient and stable vaccines.

## 2. Poliovirus surveillance and investigation of samples

Identification of poliovirus in the laboratory has had a role in the eradication programme since its inception, with detection of viruses present in stool and environmental samples acting as the major surveillance tool.

### 2.1 AFP surveillance

Poliovirus surveillance has traditionally relied on detection and sequencing of poliovirus from stool samples taken from patients with AFP. Preceding the global eradication drive the Pan-American Health Organization recommended reporting all AFP cases in children under 15 years of age and in adults where poliovirus was the suspected cause of paralysis to ensure the detection of paralytic cases (Pan American Health Organization 1987). This was incorporated into the global standards of polio reporting adopted by the GPEI. As AFP has several potential viral and non-viral causes, the overall AFP reporting rate is used as an indicator of the sensitivity of the surveillance network to detect poliomyelitis. Gaps in surveillance are a major issue for certification of eradication as poliovirus can circulate undetected for a long time in areas with weak surveillance systems.

As only one in 200 − 1,000 individuals infected with poliovirus is paralysed, the majority of infections are asymptomatic or result in mild illness and are not detected by AFP surveillance. Analysis of the genetic relatedness of polioviruses isolated from AFP cases gives an indication of the sensitivity of AFP surveillance, with viruses found to be distantly genetically related to others referred to as ‘orphan’ viruses, which are indicative of virus circulation without detection. In Nigeria, a country removed from the endemic list in September 2015, cases of WPV were detected in 2016 that were directly linked to isolates from virus circulating in the same region in 2011 (World Health Organization 2015; Nnadi et al. 2017). This suggests that poliovirus was able to circulate undetected for over 4 years whilst conflict in the north-east of the country prevented adequate AFP surveillance. Highly divergent viruses have also been detected in other settings. For example, in a study in Pakistan, 7.7 per cent of AFP isolates collected between 2011 and 2013 were orphan viruses with >1.5 per cent divergence from previously collected sequences, suggesting substantial gaps in surveillance (Cowger et al. 2017).

### 2.2 Environmental surveillance

As the incidence of paralytic polio has fallen globally, sampling of sewage networks to look for the virus has expanded; especially in countries with continuing WPV1 transmission or long-term transmission of VDPVs. The majority (>99%) of poliovirus infections are asymptomatic and do not result in paralysis, and so are not detected by AFP surveillance, but poliovirus shed in stool by infected individuals can still be detected in environmental (sewage) samples. Environmental surveillance (ES) has been shown to be more sensitive than AFP surveillance in areas with convergent sewage networks (Kroiss et al. 2018; O’Reilly et al. 2018). For example, inclusion of ES samples in Pakistan reduced the proportion of AFP isolates found to be orphans from 7.7 to 4.9 per cent (Cowger et al. 2017). Work is ongoing to target these environmental sampling locations to areas where they are likely to have the most impact by investigating catchment areas and sewage conditions.

Sewage sampling is efficient as it allows fewer resources to be used to survey a larger population of possibly affected individuals than direct door to door or local stool surveys. A study in 1991 in Cartagena, Colombia was able to directly sample stool from 196 children in 3 days in a neighbourhood with approximately 5,300 children. Seventy per cent of households in this neighbourhood were served by the sewage network and in the same time period forty-two sewage samples were taken. These samples represent repeat samples of the same 70 per cent of the population but much more complete sampling than the 3.6 per cent coverage with stool surveys. When comparing rates of poliovirus detection, 21 per cent of sewage samples were positive compared with 8 per cent of stool samples (Tambini et al. 1993).

Despite the utility of poliovirus ES, it is not without challenges. Poliovirus survival in the environment is highly variable, with a 90 per cent infectivity loss observed in 26 days in sewage at 23°C (Dowdle and Birmingham 1997). This affects virus detection in cell culture but not necessarily when direct molecular sequencing methods are used. In tropical regions, where poliovirus surveillance is often carried out, the high ambient temperature and bacterial content of the wastewater can further decrease the infective half-life of environmental poliovirus (Pan American Health Organization 2006).

### 2.3 Laboratory testing

The WHO recommended protocol for detection of poliovirus in stool or ES samples is to isolate the virus using two cell lines, one murine cell line (L20B) that is genetically modified to express the human poliovirus receptor (CD155) and selectively grows poliovirus, and one human rhabdomyosarcoma (RD) cell line which is susceptible to a broad range of enteroviruses (Vaccine Assessment and Monitoring of the Department of Vaccines and Biologicals 2003; Kroiss et al. 2018). Viruses which grow on the RD cells but not when cross-passaged on L20B cells are classed non-polio enteroviruses and typically not investigated further. Serotype and intratype (vaccine vs other strains) for any polioviruses isolated on L20B cells is determined by quantitative PCR followed by genetic sequencing (World Health Organization 2004a). Currently, the time from sample arrival at the laboratory and generation of a genetic sequence required to confirm detection is 2 − 3 weeks, although several activities are aiming to reduce this time by using direct molecular detection methods. For example, the development of sensitive PCR protocols and next generation sequencing (NGS) methods could allow both rapid detection and highly specific typing. Viral RNA can be directly extracted from both stool and ES samples, with PCR amplification and sequencing library preparation performed in as little as 2 days (Victoria et al. 2009; Majumdar et al. 2018). PCR primers targeting conserved genomic regions provide detection width, whilst variable regions between the primers allow high resolution typing (Kilpatrick et al. 2011). Furthermore, multiple polioviruses can be detected simultaneously, with NGS reads corresponding to the mixture of viruses contained in the sample.

## 3. Investigation of transmission

### 3.1 Poliovirus evolution: molecular clock and recombination

**Figure 1.**
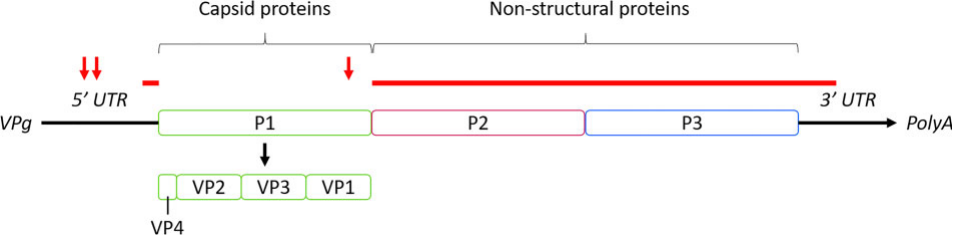
Simplified structure of the poliovirus genome adapted from Semler (2004). This diagram highlights the position of the VP1 region of the genome, routinely sequenced for typing purposes, and shows the relative positions of the other major regions mentioned in the text. Red bars show major recombinant regions determined by Stern et al. and arrows the positions of the three gatekeeper mutations for reversion to virulence in serotype two vaccine-derived polioviruses (Stern et al. 2017).

The 906-nucleotide VP1 region of the poliovirus genome encoding a portion of the viral capsid is used for typing and is the only section of the genome routinely sequenced (Fig. 1). VP1 contributes to the major neutralisation sites and amino acid residues on the poliovirus surface, making it highly representative of the interaction of poliovirus with the external environment and host cells (Minor et al. 1983; De et al. 1995; Oberste et al. 1999; Chen et al. 2013; Shaw et al. 2018). Using only this region of the genome it is possible to identify poliovirus amongst other enteroviruses and differentiate between the three serotypes (Oberste et al. 1999). Poliovirus has a consistently high number of mutations due to its highly error-prone RNA polymerase, which results in a substitution rate on the magnitude of 10^−2^ nucleotide changes per site per year in the VP1 region (Jorba et al. 2008). Mutations that are fixed in VP1 are usually synonymous as a result of selective constraints on non-synonymous mutations that change the capsid structure. Therefore, they accrue according to a molecular clock, allowing robust phylogenetic trees to be constructed and dates of emergence of new lineages to be estimated (Gavrilin et al. 2000; Jorba et al. 2008). VP1 sequences can therefore be used to investigate poliovirus transmission. Current determination of clusters of transmission follows the US Centers for Disease Control and Prevention guidance with differentiation between circulating viruses based on sequence identity. New clusters are added whenever a detected VP1 sequence is 5 per cent divergent from previously collected samples (Jorba 2016). Having an indication of the relatedness of viral sequences is a good marker of the progress towards eradication. A reduction in the number of genetic clusters can be indicative of the impact of vaccination campaigns.

In addition to having a high mutation rate, circulating polioviruses frequently recombine with other species C enteroviruses (Bessaud et al. 2016). Much of this recombination occurs in the non-structural regions of the genome during coinfections, meaning recombination does not affect the makeup of the poliovirus surface antigens or the inference of transmission routes from the VP1 sequence. Several projects have been carried out using full genome sequence data to map recombination sites in the genome (Duggal et al. 1997; Runckel et al. 2013; Holmblat et al. 2014; Stern et al. 2017). These primarily occur on the boundary between the 5′ UTR and the P1 capsid encoding region and between P1 and the P2/P3 non-structural regions (Fig. 1). Although this occurs in both wild and VDPV in circulation, it is of greater concern in vaccine and vaccine-derived strains due to the loss, through recombination, of attenuating mutations and reversion to a more transmissible pathogenic form.

### 3.2 Using virus sequences to reconstruct virus spread

**Figure 2.**
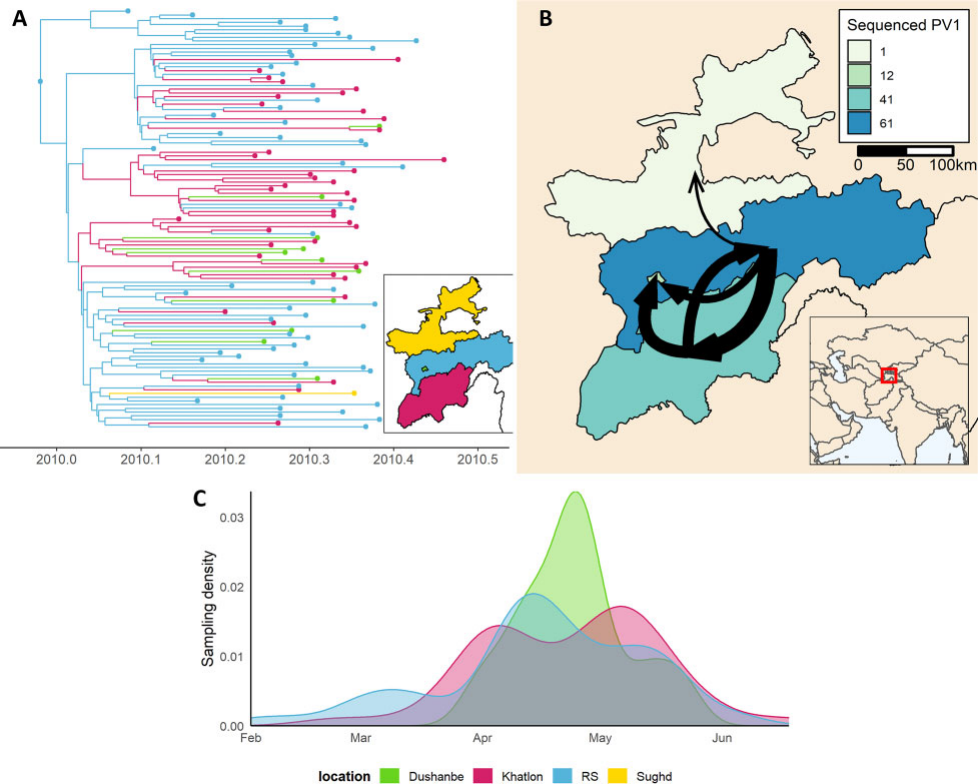
Movement of poliovirus in the west of Tajikistan during the 2010 WPV1 outbreak inferred from a discrete trait analysis of 115 publicly available sequences (Yakovenko et al. 2014). (A) Inferred phylogenetic tree for the poliovirus sequenced in the three regions in 2010. The *x*-axis is given in decimal years inferred from the clock rate and sampling date of the sequences at the tips of the phylogeny. The inset map shows the location of the four regions in western Tajikistan. (B) Inferred movement events and number of sequenced poliovirus stool samples per region. The breadth of the arrows indicates the number of inferred movement events. The end points of the arrows represent the centroid of each region and not the exact location of reported events. The inset map shows the location of the study area in the wider context of Central and South Asia. (C) Sampling density of sequences over time in the regions shown in (A). Sughd has only one sequence collected on 10 May 2010 so does not feature in this density plot.

Investigation of the genetic relatedness of isolates through VP1 sequencing has been critical in distinguishing areas with persistent transmission from those suffering repeated introductions of poliovirus. Application of methods from phylogeography has allowed complex virus movement dynamics to be unravelled, and sources and sinks of infection to be identified. One of these methods is based on the discrete trait analysis (DTA), which can provide information on the geographic history of viral movement and associated rates. Starting from geolocated virus sequences, this approach allows inference of the location of internal nodes in phylogenetic trees. DTA has previously been applied to inform viral diffusion for several infectious diseases including rabies, influenza and Ebola (Lemey et al. 2014; Dudas et al. 2017; Yang et al. 2019). An illustrative example with a small dataset is presented here using data from the 2010 WPV1 Tajikistan outbreak, available on GenBank (KC880365–KC880521) (Yakovenko et al. 2014) (Fig. 2). Case reporting from this outbreak shows a large number of cases in both the region of Republican Subordination (RS) and Khatlon, as well as a number of temporally clustered cases in the capital city Dushanbe (separated from RS for this analysis). When a phylogeographic model that incorporates sampling location and time is implemented, it becomes clear that transmission within Tajikistan likely originates in RS, which also supports the majority of virus transmission. All sequenced cases in Dushanbe are inferred to be imported with no onwards transmission within the city or to other regions. Earlier phylogenetic analysis also points to a single importation of wild-type one poliovirus into Tajikistan from India and onwards transmission, which cannot be inferred from case data alone (Yakovenko et al. 2014).

Ongoing analyses using the DTA approach have been applied to WPV1 sequence data from Pakistan and Afghanistan, where WPV1 persists with complex circulation dynamics and geographical spread due to mobile and hard to reach populations. These preliminary analyses have identified cycles of transmission between the south-west of each country (Kandahar/Quetta and Karachi). They also highlight repeated introductions of virus from the tribal regions (between Islamabad and Kabul) into the Punjab province of Pakistan and show uninterrupted virus circulation within these tribal regions. Analysis of the distribution of clusters of transmission without formal DTA analysis also highlights these three, key, high-burden regions but does not construct probable ancestral locations or virus movements (Akhtar et al. 2019).

Phylogenetic visualisations that split trees into clusters of transmission based on those presented by Dudas et al. for the 2014 Ebola outbreak and interactive phylogenetic trees using the Nextstrain platform have also been developed, and are provided in internal WHO reports, but are not yet publicly available (Dudas et al. 2017; Hadfield et al. 2018). Regular updates of the polio surveillance sequence data using these new visualisation tools may provide insights into changes in the polio epidemiology, which can in turn guide the implementation of more strategic supplementary immunisation activities. These analyses may be particularly relevant to halt the remaining circulation of wild poliovirus.

Given the declaration of polio as a PHEIC and the value of poliovirus genetic sequence data to interpreting its epidemiology, there is an urgency to share poliovirus sequences and phylogenetic trees more rapidly and more widely to allow broad scrutiny by scientists and policy makers. This is being pursued with the planned creation of a global polio nucleotide sequence database (PONS) by the US CDC.

## 4. Vaccine-derived polioviruses

Live-attenuated OPV strains replicate in the gut to induce both local mucosal and systemic immunity, the former being important to prevent transmission of wild viruses. This is a major benefit over injected IPV, which provides systemic immunity against paralysis but limited intestinal mucosal immunity. Replication of OPV strains is, however, associated with virus shedding and transmission to contacts. Selection for loss of the attenuating mutations which reduce OPV virulence occurs during virus replication through mutation and recombination. In rare cases, this can lead the vaccine virus to regain pathogenicity and transmissibility similar to that seen in wild-type polioviruses. Vaccine strains are classified according to their source and their relatedness to the Sabin strain and other polioviruses. Current reporting standards define serotype two VDPVs as those viruses with at least six nucleotides difference from the Sabin strain in the VP1 region (at least ten nucleotides for serotypes one and three). Vaccine-related viruses not reaching this threshold are classified as ‘Sabin-like’. Although it is possible for Sabin-like viruses to cause poliomyelitis, this occurs much less frequently than with viruses which have crossed the threshold to be classified as VDPVs (Zurbriggen et al. 2008; Korotkova et al. 2016).

Replication and shedding of the vaccine virus in the intestine and throat can occur long term in individuals with a primary B cell immunodeficiency who have been vaccinated with OPV as they are not able to clear vaccine virus infection. These immunodeficient VDPVs (iVDPV) may pose a risk to the wider population and have required the introduction of a primary immunodeficiency surveillance programme by the GPEI (World Health Organization 2019). Long-term shedding of iVDPV can result in highly divergent viruses. For example, one individual being monitored in the UK was shedding a virus with 17.7 per cent VP1 sequence divergence from the Sabin strain in 2015, approximately 28 years after vaccination (Dunn et al. 2015).

VDPVs are classed as ambiguous (aVDPVs) when they are not linked to ongoing chains of transmission or to any known immunodeficient individuals. When linked cases are subsequently identified, many aVDPVs are re-classed as circulating VDPVs (cVDPVs). cVDPVs may cause large outbreaks of poliomyelitis and are a major threat to the eradication of poliovirus. They are likely to emerge where population immunity is low, and sanitation is poor (Pons-Salort et al. 2016a).

A number of studies have highlighted key ‘gatekeeper’ mutations required for Sabin poliovirus to regain wild-type fitness in the human intestine. Recent studies of serotype two poliovirus agree on three specific codon positions for these reversions, two in the 5′ UTR and the third in the protein coding VP1 region (Fig. 1) (Famulare et al. 2015; Stern et al. 2017), two of which were first published in 1991 (Macadam et al. 1991; Ren, Moss, and Racaniello 1991). Alongside specific point mutations, studies have found that the majority of cVDPVs are recombinant with other species C enteroviruses (Jegouic et al. 2009; Burns et al. 2013). This often provides revertant viruses with more efficient viral protease and RNA-dependent RNA polymerase. Burns et al. found that 95 per cent of cVDPV2 isolates from northern Nigeria between 2005 and 2011 had lost the major attenuating mutations in the 5′ UTR by recombination rather than by point mutation and all isolates had lost the major attenuating mutation in VP1 by recombination (Burns et al. 2013).

### 4.1 Extent of cVDPV transmission

Since the 2016 switch from trivalent to bivalent OPV, the incidence of VDPV2 outbreaks has increased with 29 VDPV2 outbreaks in 15 countries reported between January 2018 and June 2019 compared with nine in six countries in the same period in 2017 − 8 (Jorba et al. 2019). This increase in incidence is largely the result of the replacement of trivalent with bivalent OPV in routine and supplementary vaccination activities and the risk of cVDPV2 transmission associated with falling population immunity to serotype two poliovirus (Pons-Salort et al. 2016b; Blake et al. 2018). Analysis of recent cVDPV2 outbreaks has shown that they mostly emerged from mOPV2 given in response to previous outbreaks (Macklin et al. 2020). This raises the serious concern that mOPV2 is not a suitable tool for cVDPV2 outbreak response in the context of a growing global cohort of children who have not been immunised with OPV2.

**Figure 3.**
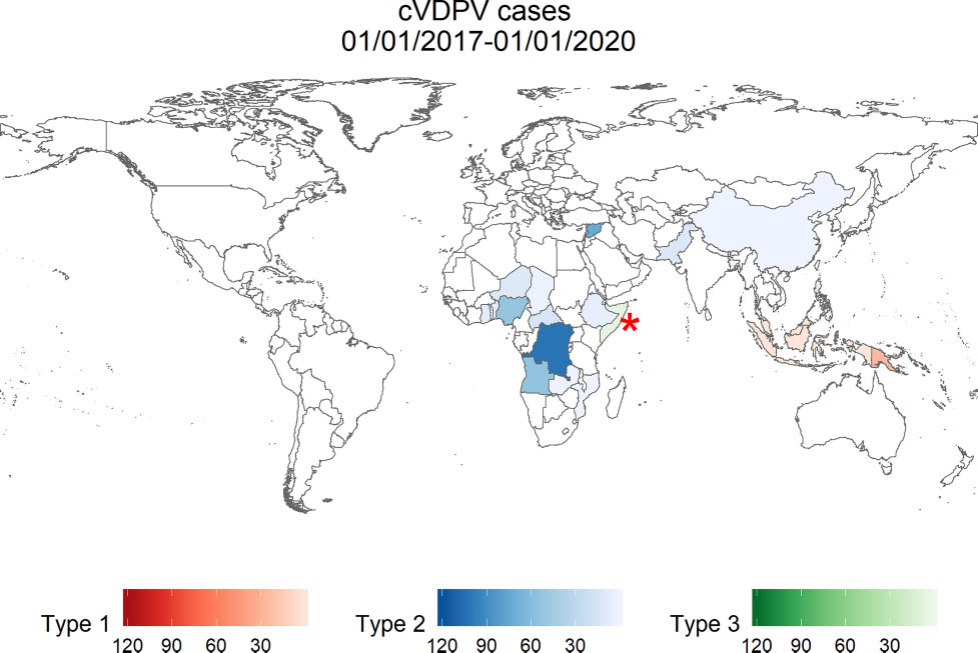
Countries reporting circulating vaccine-derived poliovirus (cVDPV) between 1 January 2017 and 1 January 2020. These countries are shaded according to the number of paralysis cases reported over this time period. The red, blue, and green represent serotype one, two, and three VDPVs, respectively. Data from the polio information system of the global polio eradication initiative (World Health Organization 2014c). *Somalia has reported both serotype 2 cVDPV (eight cases) and serotype three (six cases) in the time period covered by this plot.

cVDPV cases have been reported in fifteen countries between January 2017 and January 2020 (Fig. 3) (World Health Organization 2014c; Greene et al. 2019; Thornton 2019). Using genetic data, it is possible to infer which of these cases are due to local transmission and which are more likely to be linked to chains of cVDPV transmission in other countries. The polio eradication programme has been able to link cVDPV2 cases in Benin, Cameroon, Ghana, and Niger to ongoing circulation in Northern Nigeria, whereas cases in Angola and Democratic Republic of the Congo were found to be the result of unlinked local emergences (Jorba et al. 2019). Understanding which cases are due to under-vaccinated local populations and which are due to international spread is important to determine the correct vaccine response and to investigate the cause of the outbreak. In addition, genetic divergence from the originating Sabin strain is used to determine how long a virus has been circulating at the time of detection which influences the scale of the response mounted. Some studies have highlighted a faster mutation rate of sites under strong positive selection pressure early in VDPV emergence. This leads to a slight acceleration in the molecular clock corresponding to a shortening of inferred dates of divergence by about 30 days (Famulare et al. 2015; Stern et al. 2017).

### 4.2 New strategies to control cVDPV outbreaks

When the live-attenuated Sabin OPV was in development almost 70 years ago, potential vaccine strains were selected empirically based on their immunogenicity and neuropathogenicity in animals prior to testing in clinical trials. Current projects to update vaccines can now be informed by our knowledge of the structure and function of the genome. More genetically stable OPVs are in development with genomes edited to reduce the risk of reversion to neurovirulence (Knowlson et al. 2015). Human phase one trials of novel OPV2 candidate vaccines were carried out in 2018 with improvements to the stability of the major attenuating mutation in the 5′ UTR (Van Damme et al. 2019). Each candidate also had further mutations to inhibit recombination and reduce replicative fitness, respectively. Small quantities of novel OPV2 vaccines are expected to enter the vaccination programme for emergency use in mid-2020 (Jorba et al. 2019; Van Damme et al. 2019). These may become a key tool for the programme to stop the current VDPV2 outbreaks while avoiding seeding new ones. At this critical point post-switch, the reintroduction of routine tOPV vaccination is also being debated as a possible solution to control the increasing number of serotype 2 vaccine-derived cases reported. Re-introduction of tOPV would represent a major step back in the polio eradication campaign, away from the phase out of current live vaccines, but would also reintroduce type two immunity in children, helping to control future VDPV2 outbreaks.

## 5. Conclusion

With an upsurge in the number of wild poliovirus cases in 2019, and nine countries affected by ongoing VDPV outbreaks at the beginning of 2020, the GPEI is at a critical stage. Recent improvements in genetic sequencing and analysis techniques are helping support efforts to achieve a polio-free world. The main current lines of research include the development of direct detection and sequencing protocols that allow rapid detection and typing of poliovirus from stool and environmental samples (thus reducing the time from sample collection to reporting); the implementation of intuitive pipelines for bioinformatics and genetic analysis that speed and standardise the production of genetic data; the implementation of phylogeographic analysis and development of visualisations tools that allow analysis and interpretation of the genetic data by planners and policymakers; and the production of new oral vaccines based on newly engineered strains that have a lower risk to revert and regain virulence.

## Data availability

No new data is presented in this article. An illustrative example is presented using publicly accessible data from GenBank (KC880365–KC880521).
